# Current updates in machine learning in the prediction of therapeutic outcome of hepatocellular carcinoma: what should we know?

**DOI:** 10.1186/s13244-021-00977-9

**Published:** 2021-03-06

**Authors:** Zhi-Min Zou, De-Hua Chang, Hui Liu, Yu-Dong Xiao

**Affiliations:** 1 Department of Radiology, The Second Xiangya Hospital of Central South University, No.139 Middle Renmin Road, Changsha, 410011 China; 2 Department of Diagnostic and Interventional Radiology, University Hospital Heidelberg, 69120 Heidelberg, Germany

**Keywords:** Hepatocellular carcinoma, Machine learning, Predictive, Modality

## Abstract

With the development of machine learning (ML) algorithms, a growing number of predictive models have been established for predicting the therapeutic outcome of patients with hepatocellular carcinoma (HCC) after various treatment modalities. By using the different combinations of clinical and radiological variables, ML algorithms can simulate human learning to detect hidden patterns within the data and play a critical role in artificial intelligence techniques. Compared to traditional statistical methods, ML methods have greater predictive effects. ML algorithms are widely applied in nearly all steps of model establishment, such as imaging feature extraction, predictive factor classification, and model development. Therefore, this review presents the literature pertaining to ML algorithms and aims to summarize the strengths and limitations of ML, as well as its potential value in prognostic prediction, after various treatment modalities for HCC.

## Key points

1. To highlight the effectiveness of machine learning algorithm on the prediction of therapeutic outcome for hepatocellular carcinoma after various treatment modalities
2. To illustrate the advantages and disadvantages of each machine learning algorithm
3. To familiarize the challenges of selecting a machine learning algorithm when creating a model

## Introduction

Hepatocellular carcinoma (HCC) is an aggressive tumor which remains the second-most frequent cause of cancer death worldwide [1–3]. According to the different statuses of patients with HCC, several guidelines [4–7] recommend various treatment strategies. Due to the aggressive biological behavior of HCC, recurrence is not uncommon. Therefore, it is essential to predict therapeutic outcomes prior to treatment so that physicians can design a personalized therapeutic strategy for each patient. The conventional process of model establishment is selecting the appropriate predictors, utilizing them for statistical analysis and ultimately deriving a multivariate predictive model [8–12]. However, predictive models developed by traditional statistical methods, such as the logistic regression (LR) model and Cox proportional hazards model, are not reliable because the factors included in the models are too simple and utilize a low evidence level. Machine learning (ML) is a powerful tool for generating high-level medical features or combining quantitative radiomic parameters with efficient algorithms [13–16]. ML algorithms simulate human learning to detect hidden patterns within HCC therapeutic data that are clearer than those derived from traditional statistical methods. With this in mind, ML algorithm has been used in many studies to predict the therapeutic outcome of HCC patients. Thus, in this review, the advantages and disadvantages of each ML algorithm are clarified, and relevant literature on the prediction of therapeutic outcomes after various treatment modalities for HCC is described.

### Advantages and disadvantages of the ML algorithm

ML algorithms have several advantages over traditional statistical methods. First, traditional statistical methods can only process the variables that have a linear relationship with the outcome [12], whereas ML algorithms have the ability to process nonlinear data. Second, ML algorithms can learn from existing data to find novel patterns between variables and generate predictions [17–20]. Third, the ML model may contain more variables [21, 22] since the variables do not simply rely on the selection of traditional statistical methods [23–25]. Last, ML methods can process big data at a high speed.

Although ML algorithms are increasingly used, the disadvantages of ML algorithms should be kept in mind. First, the current ML methods are still not readily available for clinical practice, and the design of the ML model is not standard. Second, the lack of perfect generalization capability is still a common issue in clinical practice. The detailed advantages and disadvantages of the ML algorithm are listed in Table 1.

**Table 1:** Advantages and disadvantages of ML algorithm

	Content
Advantages	It can process a big data It can process a nonlinear data It can be used to select meaningful predictive variables and extract radiomic variables It usually has higher predictive performance than traditional statistical model
Disadvantages	The accurate selection of ML algorithms is a challenge to establish a predictive model The design of ML predictive models lacks standards It is difficult to identify the process of ML model development due to the existence of “black box” The generalization ability of the established model needs to be further confirmed in validation cohort

*ML* machine learning

### ML models in the prediction of therapeutic outcomes for HCC

With the development of ML algorithms, a growing number of studies have developed prognostic predictive models for HCC using the ML method. Therefore, understanding how ML works is essential. In this section, various ML models are introduced.

### Neural networks

**Fig. 1:**
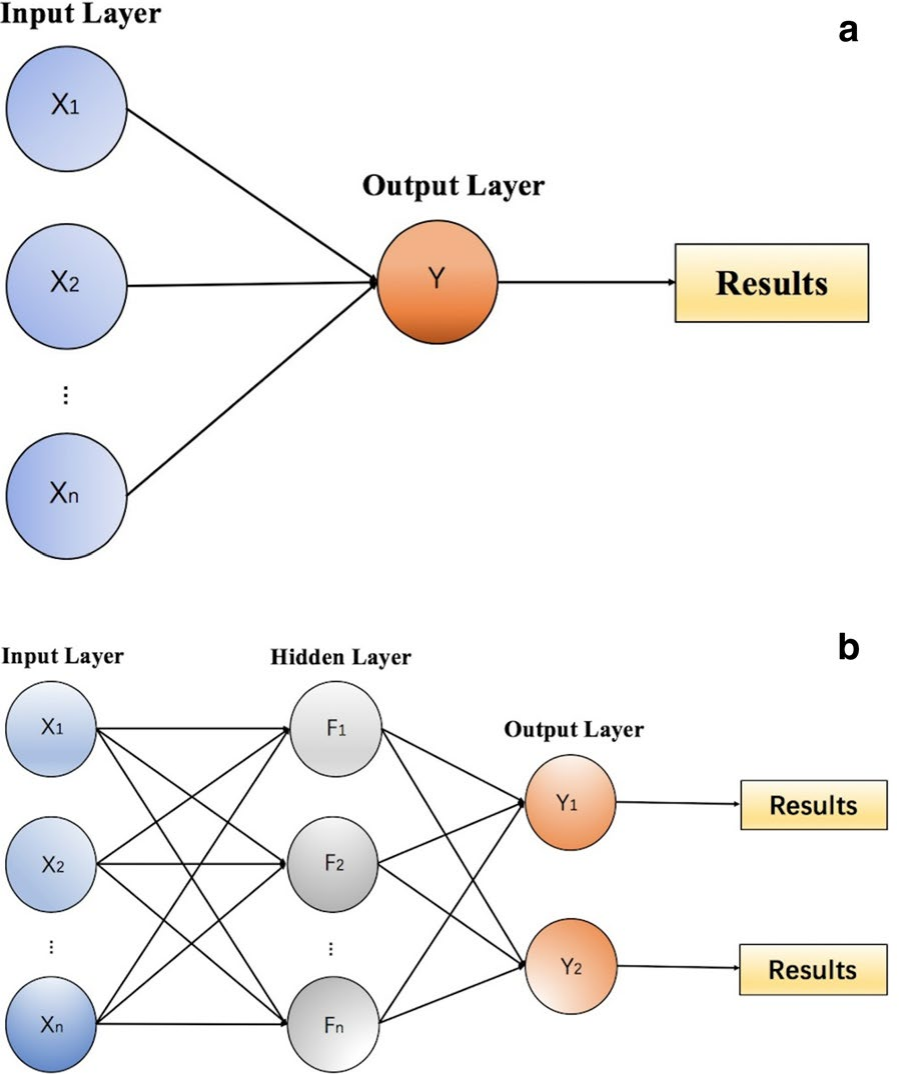
Schematic diagram of an artificial neural network (ANN). **a** shows a schematic diagram of a perceptron. It is the simplest model of an ANN and only includes an input layer and an output layer. In the perceptron, the input feature parameters are directly converted to the output results through the weight between the input and output. **b** shows a schematic diagram of a 3-layer ANN (also called a multilayer perception). The first layer is the input layer, corresponding to the input feature parameters (X); the middle is the hidden layer, which uses a composite function to achieve the abstraction for input features so that the input can be better divided linearly; the last is the output layer, where the number of categories to be classified determines the number of neurons in this layer, and its output value (Y) is the predictive value of the ANN

Neural networks are a classic ML method that simulates human brain neural networks. The most widely used neural networks are artificial neural networks (ANNs) and deep neural networks (DNNs). ANN [26] is one of the earliest neural network models and can be divided into three components: an input layer, a hidden layer, and an output layer. The ANN model can include a perceptron or a multilayer perceptron (MLP) (Fig. 1), with or without a hidden layer. However, ANNs cannot directly deal with medical imaging. With the development of deep learning, DNNs are widely used in establishing models [27]. Convolutional neural networks (CNNs) [28, 29] are one of the most common DNNs that can automatically identify and segment medical imaging. Another type of DNN is the recurrent neural network (RNN). However, RNNs are limited in HCC prognostic studies because the RNN algorithm cannot process data over a large time span.

### Support vector machines

**Fig. 2:**
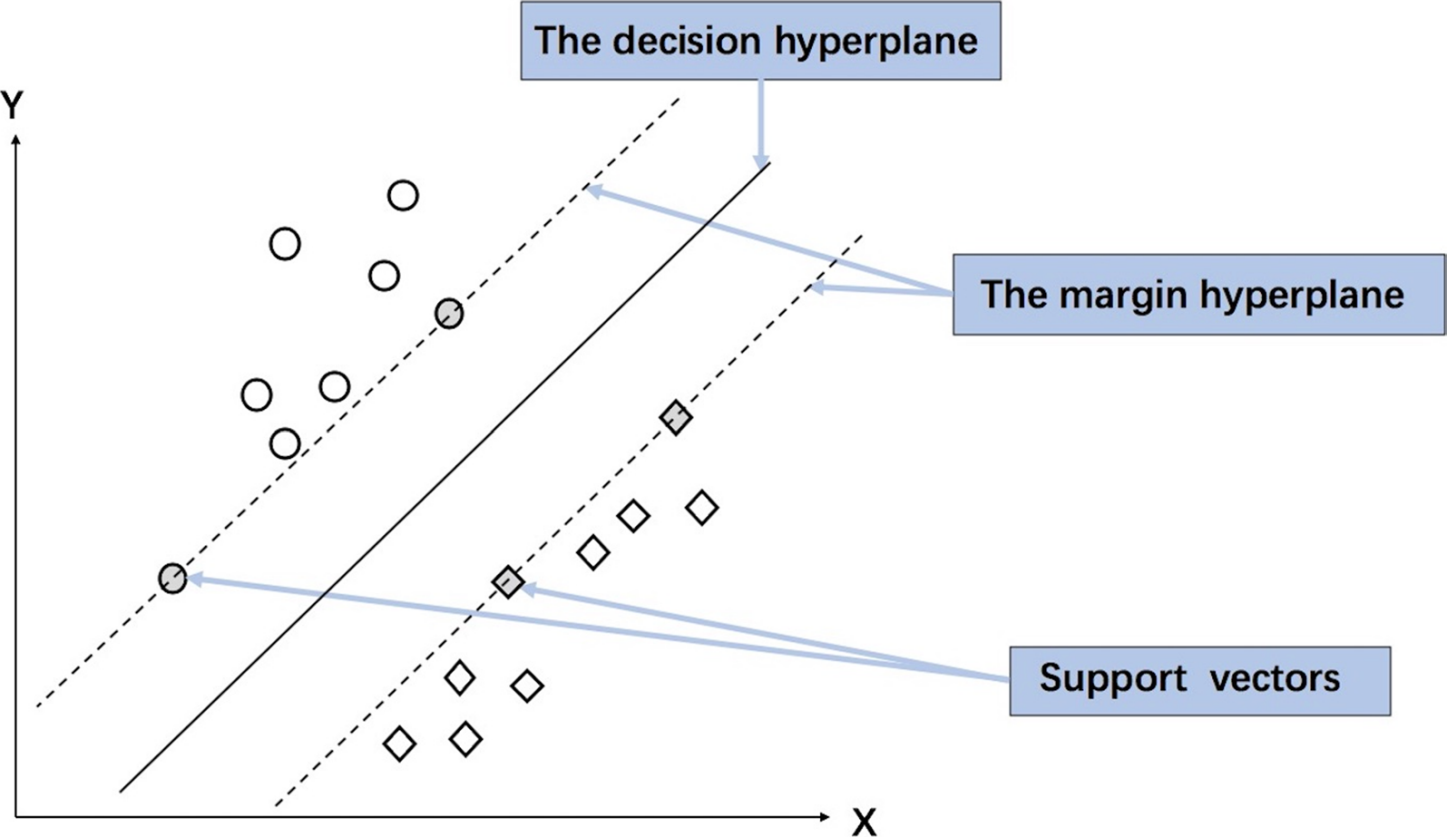
Schematic diagram of a support vector machine (SVM). Although a decision hyperplane with maximal margin separates every sample into two classes, the support vector is the sample point on the margin hyperplane, which is the largest margin of classification under the constraints. The final SVM model is only related to support vectors

A support vector machine (SVM) [30] is a type of two-category model aimed at finding the optimal separating hyperplane with the largest distance to the support vector of any class (Fig. 2). Due to the hyperplane concept, SVM is often used for the selection of parameters for which the parameters are selected by the correlation to the results. However, SVMs are only applied in studies with small sample sizes, as the number of support vectors in large datasets is still very big, which may increase the complexity and training time of SVM algorithms.

### Decision tree and random forest

**Fig. 3:**
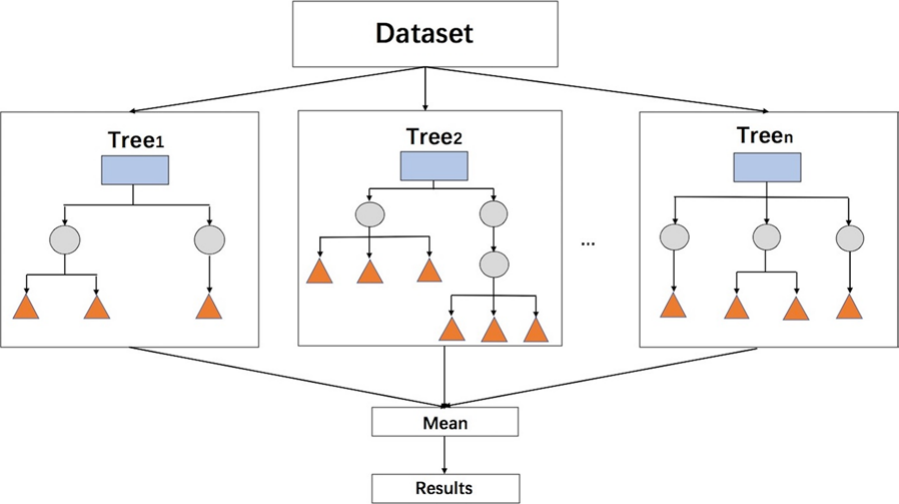
Schematic diagram of random forests (RFs). An RF is an ensemble learning approach that consists of multiple decision trees. In the process of training the RF model, each decision tree is trained in sequence, and the training samples of each tree are extracted from the original datasets by a randomized sampling method. The features used in each decision tree are also obtained by random sampling. The joint prediction of multiple decision trees improves the accuracy of the RF model

The decision tree (DT) [31] is easy to understand and adopts a form of yes or no question and comprises a root node, parent node and leaf node/terminal node. Unfortunately, with the increasing complexity of the DT model, the predictive value inevitability decreases. Random forest (RF) [32] represents an ensemble learning approach of multiple unique DTs, which is designed to increase the predictive performance (Fig. 3). In the training procedure, the bootstrap sampling method is used to construct each tree based on randomized samples and features from the original dataset, and the final result of the RF is the average prediction of each tree. In most ML algorithms, RFs have the highest predictive performance [33]. Ishwaran et al. [34] designed random survival forests (RSFs), an extension of RFs to right-censored survival data. However, due to the complexity of RF, the processing requires more time for training the model compared with other ML algorithms.

### Bayesian networks

**Fig. 4:**
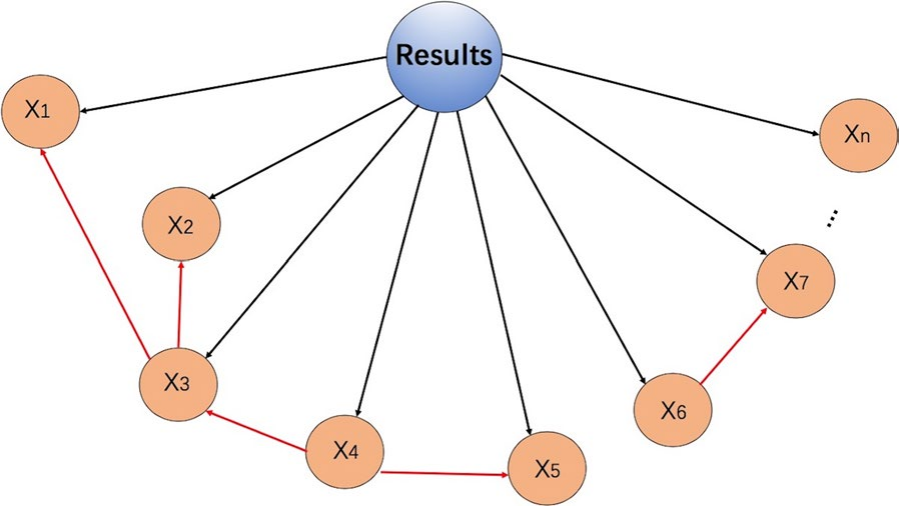
Schematic diagram of a Bayesian network (BN). A BN is a directed acyclic graph that consists of nodes, edges and conditional probability. The nodes represent random parameters, and the directed edges between the nodes represent conditional dependencies (from the parent node to its child nodes). The interdependence between the nodes is expressed with conditional probability, and the classification result is the class with the highest conditional probability

Bayesian networks (BNs) [35] are different from most ML algorithms. A BN is an extension of Bayes’ theorem and presents the causality under each variable via a directed acyclic graph (Fig. 4). Therefore, those algorithms can visualize information. BNs have been applied to analyze predictors for survival in postsurgical HCC patients through conditional probability tables (CPTs) [36]. However, the relationship between each variable in the BN model is not always clear, which leads to low accuracy.

## Methods

**Fig. 5:**
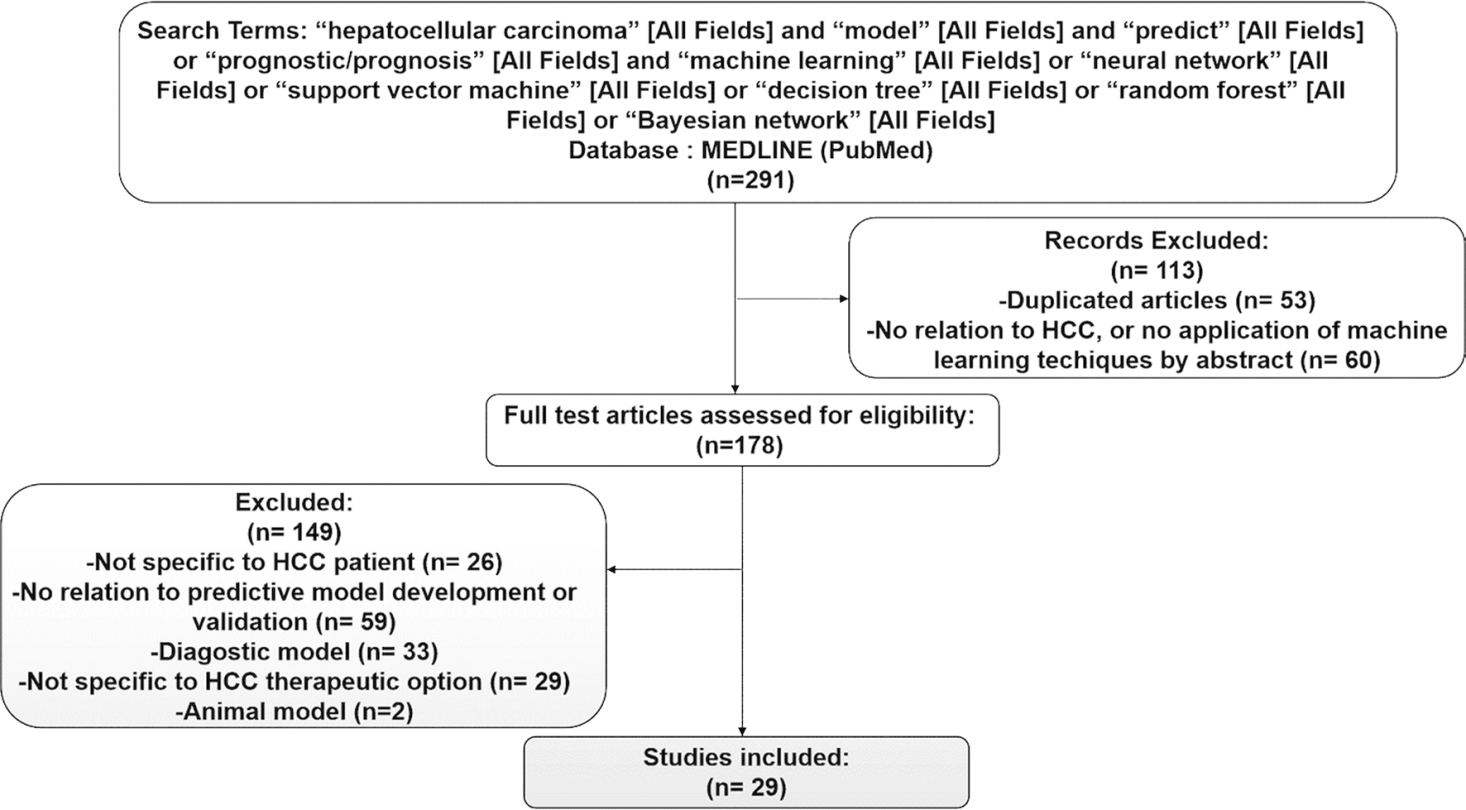
Flowchart of the search strategy and selection of studies for inclusion

A search of PubMed was conducted for a prognostic predictive model for HCC published from January 1995 to May 2020. The following search algorithm was created: “hepatocellular carcinoma” and “model” and “predict” or “prognostic/prognosis” and “machine learning” or “neural network” or “support vector machine” or “decision tree” or “random forest” or “Bayesian network”. Initially, a total of 291 relevant research articles were searched, and the literature selection process is shown in Fig. 5. Ultimately, 29 articles were enrolled in the final analysis.

### Prediction of therapeutic outcomes by various treatment modalities

There are various treatment options for HCC. Surgical resection, ablative therapy, and liver transplantation (LT) are potentially curative treatments, and transarterial chemoembolization (TACE) and sorafenib are palliative treatments [37–40]. Due to the poor prognosis of HCC patients, it is essential to create a suitable predictive model for predicting therapeutic outcomes prior to treatment. In this section, the current updates of ML algorithms are reviewed for various treatment modalities in HCC patients.

### Surgical resection

Partial hepatectomy remains the mainstay of curative treatment in the early stage of HCC. Intrahepatic recurrence of HCC after surgical resection is the major cause of death, as the incidence rate is approximately 70% at 5 years [41, 42]. Therefore, an accurate prediction of prognosis prior to resection is crucial.

In previous studies, the authors developed a predictive ANN model [43–47] to predict therapeutic outcomes after surgical resection, and the ANN model was verified to be superior to the LR model and Cox proportional hazards regression model. Unfortunately, the ANN model cannot be used to select variables, which may decrease the predictive accuracy when some potentially clinically meaningful variables are overlooked. Similar to ANN, the BN model [19, 36] also cannot be used to select variables. The predictive variables within the BN model are based on the clinician’s experience and knowledge, and the associated relationship between variables and outcome is not always clear; therefore, the performance of the BN model is generally confusing. Unlike ANN and BN, RF and SVM can be used to either select variables or develop models [48–54]. Wang et al. [52] used the RF algorithm to select 30 radiomic features from 3144 MR texture features and developed a predictive RSF model for the 5-year survival of HCC following surgical resection with an area under curve (AUC) of 0.980. In addition, Liao et al. developed an RF model [53] based on 46 features from whole slide images (WSIs), and the results showed comparable accuracy to the TNM staging system in predicting the prognosis of HCC patients after surgical resection. However, it should be noted that the sample size in the clinical study of HCC is usually small, and the SVM model is theoretically more suitable than other models. Xu et al. used an immunohistochemistry (IHC)-based SVM algorithm [48] to predict the recurrence of 336 HCC patients after surgical resection. The SVM model finally selected 8 features from 49 features and had an accuracy of 82.1%. In comparison to the abovementioned ML algorithms, the CNN algorithm has great convenience in establishing predictive models because they can be used not only to segment imaging but also to select parameters and to develop models [23, 55]. Wang et al. [23] used the CNN algorithm to extract high-level temporal and spatial features from multiphase CT imaging using an automatic mode, which showed high efficacy with an AUC of 0.825 for predicting the early recurrence of HCC. Nevertheless, the automatic mode based on the CNN algorithm requires high computational power and thus has limited use. The relevant papers are listed in Table 2.

**Table 2:** Characteristics of ML-based predictive model of HCC patients after hepatectomy

Author	Study type	No. of patients	Model	Outcomes	AUC/C-index	Conclusion
Hamamoto [43], 1995	Retrospective Single center	65	ANN	Death	–	In the study for predicting the died of hepatic dysfunction, ANN predicted the outcome of 11 patients in the validation group and achieved the accuracy of 100%
Ho [44], 2012	Retrospective Multicenter	427	ANN and DT	1,3,5-year DFS	D: 0.977 and 0.734 (1-year) 0.989 and 0.825 (3-year) 0.963 and 0.675 (5-year) V: 0.777 and 0.718 (1-year) 0.774 and 0.561 (3-year) 0.864 and 0.627 (5-year)	The ANN outperforms DT in predicting DFS in post-surgical HCC patients
Xu [48], 2012	Retrospective Multicenter	336	SVM	RR	–	The SVM based on IHC features could identify HCC patients who are easily recurrence after surgery, and the predictive accuracy of SVM was 66.5%
Chiu [45], 2013	Retrospective Multicenter	434	ANN	1,3,5-year survival	D: 0.980 (1-year) 0.989 (3-year) 0.993 (5-year) V: 0.875 (1-year) 0.798 (3-year) 0.810 (5-year)	The ANN model can process a greater number of predictors and had better accuracy than the traditional LR model
Qiao [46], 2014	Prospective Multicenter	725	ANN	5-year survival	D: 0.855 V: 0.829	The ANN model outperforms both Cox and other staging systems in predicting survival in HCC patients who have received surgical resection
Cai [36], 2015	Retrospective Single center	299	BN	10-month survival	–	The BN model had 67.2% of accuracy to classify the survival time of post-surgical HCC patients
Akai [49], 2018	Retrospective Single center	127	RSF	DFS, OS	0.611 0.701	RSF can predict the individual risk for each patient on DFS and OS
Wang [23], 2019	Retrospective Single center	167	DCNN	RR	0.825	Combined clinical information and radiomics features can effectively predict early recurrence of HCC patients
Kim [50], 2019	Retrospective Single center	167	RSF1* RSF2** RSF3***	Early recurrence Lately recurrence	Early recurrence: 0.671(RSF1) 0.679(RSF2) 0.707(RSF3) Early recurrence: 0.737(RSF1) 0.622(RSF2) 0.716(RSF3)	Compared to another two RSF models, combined clinicopathologic-radiomic RSF model achieved the highest predictive power for the recurrence within 2 years after surgery of HCC, and has fair predictive performance for lately recurrence
Xu [19], 2019	Retrospective Multicenter	1139	SVM RF BN	RR	–	The accuracy of SVM, RF and BN model was 0.46, 0.48 and 0.56, respectively, in validation group form another independent institution. The BN model could contribute to HCC recurrence research
Mai [47], 2020	Retrospective Single center	353	ANN	PHLF	0.880(D) 0.876(V)	The risk of severe PHIF in HCC patients after surgery based on ANN model, can be accurately divided into 3 groups
Saillard [55], 2020	Retrospective Multicenter	522	CNN1# CNN2##	OS	D: 0.75(CNN1) 0.78(CNN2) V: 0.68(CNN1) 0.70(CNN2)	Two CNN models based on histological features form WSIs performed well for predicting OS of HCC patients after surgery, and both CNN models outperformed the CS that the score included the relevant clinical, biological and pathological features
Schoenberg [51], 2020	Retrospective Single center	180	RF	DFS	D: 0.766(0.627–0.904) V: 0.788(0.658–0.919)	RF model based on clinical and laboratory variables, can accurately predict DFS after surgery of HCC
Wang [52], 2020	Retrospective Multicenter	201	RF	5-year survival	D: 0.980 V: 0.758	RAD model integrated with RF in a valid method to predict 5-year survival of post-operative HCC patients
Liao [53], 2020	Retrospective Multicenter	645	RF	1,3,5-Y survival	V1: 0.626(1-year) 0.658(3-year) 0.581(5-year) V2: 0.600(1-year) 0.595(3-year) 0.566(5-year)	RF model based on 46 histopathplogical features, was able to stratify post-surgical patients of HCC into long and short-term groups. And the RF model showed similar accuracy with TNM staging systems
Saito [54], 2020	Retrospective Multicenter	158	SVM	RR	–	The SVM model based on digital pathological images has the accuracy of 89.9% for prediction of HCC recurrence after surgery

*RSF1: clinicopathologic model using RSF; **RSF2: radiomic model using RSF; ***RSF3: combined clinicopathologic-radiomic model using RSF

^#^CNN1: convolutional neural network model with automatically method to processing WSI imaging; ^##^CNN2: convolutional neural network model with an attention mechanism for WSI annotation by pathologist

*ML* machine learning, *HCC* hepatocellular carcinoma, *AUC* area under the curve, *ANN* artificial neural network, *DT* decision tree, *DFS*disease free survival, *D* development cohort, *V* validation cohort, *SVM* support vector machine, *RR* recurrence rate, *IHC* immunohistochemistry, *LR* logistic regression, *BN* bayesian network, *RSF* recurrence-free survival, *OS* overall survival, *DCNN* deep convolutional neural network, *RF* random forest, *PHIF* posthepatectomy liver failure, *WSI* whole-slide imaging, *RAD* radiomic, *TNM* tumor node metastasis

### LT

LT is regarded as an effective therapy in HCC patients who are within the Milan criteria, with a recurrence rate of 10–15% [38, 56, 57]. Once post-LT HCC recurrence occurs, the prognosis is poor. Therefore, it is necessary to accurately identify HCC patients who will benefit from LT, thereby optimizing donor-recipient matching.

To our knowledge, ML-based analysis in predicting therapeutic outcomes for HCC after LT is rather limited. Marsh et al. [58, 59] developed an ANN model using seven clinical factors to predict the recurrence risk in HCC patients after LT, and the results showed that the discriminatory power was 70%. However, in the combination of this model and other variables, such as genotyping for microsatellite mutations/deletions (TM-GTP), the predictive performance increased from 70 to 85%. Rodriguez-Luna et al. [60] externally validated the ANN/TM-GTP model, and the discriminatory power was 89.5%, while the sample size in the external validation cohort was too small, comprising only 19 patients; therefore, the predictive performance was less convincing. A multicenter study conducted by Nam et al. showed more convincing results [24] because they developed a DNN model and included a relatively large sample size, in which the training cohort was 563 and the validation cohort was 214. Nevertheless, the predictive model should be based not only on the characteristics of receipts but also on donors. Therefore, precise receipt-donor matching is crucial to develop a predictive model. Zhang et al. [61] established an MLP model by including 14 characteristics of donors as well as recipients. The results showed that the c-statistics of the specific MLPs at 1, 2, and 5 years were 0.909, 0.888, and 0.845, respectively. However, the main weakness of this MLP model is the lack of external validation, and the generalization of this model needs to be further confirmed. The relevant papers are listed in Table 3.

**Table 3:** Characteristics of ML-based predictive model of HCC patients after transplantation

Author	Study type	No. of patients	Model	Outcomes	AUC/C-statistics	Conclusion
Marsh [58], 1997	Retrospective Single center	214	ANN	1,2,3-year RR	D: 0.962 ± 0.01 (1-year) 0.944 ± 0.05 (2-year) 0.952 ± 0.04 (3-year) V: 0.962 ± 0.043 (1-year) 0.966 ± 0.025 (2-year) 0.971 ± 0.034 (3-year)	The ANN model can identify post-LT HCC patients with or without recurrence
Marsh [59], 2003	Retrospective Single center	214	ANN	1,2,3-year RR	0.98 (1-year) 0.95 (2-year) 0.96 (3-year)	The ANN has genotyping as input parameter, which is possible to predict recurrence risk of post-LT HCC
Rodriguez-Luna [60], 2005	Retrospective Single center	19	ANN	Recurrence	–	This study validates the result conducted by Marsh et al., which the model had the discrimination power of 89.5%
Zhang [61], 2012	Retrospective Single center	290	MLP	1,2,5-year survival	0.909 (1-year) 0.888 (2-year) 0.845 (5-year)	The MLP model had high accuracy to predict post-transplant mortality risk for HCC recipients
Nam [24], 2020	Retrospective Multicenter	563	DNN	Recurrence	0.75	The DNN model showed promising predictive performance and outperformed other traditional predictive model to predict HCC recurrence after LT

*ML* machine learning, *HCC* hepatocellular carcinoma, *AUC* area under the curve, *ANN* artificial neural network, *RR* recurrence rate, *D* development cohort, *V* validation cohort, *LT* liver transplantation, *MLP* multilayer perceptron, *DNN* deep neural network

### Local ablation

A string of image-guided percutaneous ablations encompasses a great variety of techniques, including radiofrequency ablation (RFA), microwave ablation (MWA), ethanol injection, and cryoablation [62–65]. As RFA is the most frequently used ablation modality for HCC [66, 67], the main topic addressed in this section is RFA. Although RFA has shown good feasibility in local tumor control for HCC, complete ablation is slightly idealistic, and the relapse rate ranges from 49 to 63% [68]. When a recurrence of HCC after ablation arises, the proliferation and invasive ability of tumors are markedly increased.

Very few studies have used ML models to predict therapeutic outcomes in HCC patients in the setting of RFA. In a small sample analysis of 83 HCC patients [69], an SVM model was used to analyze the relationship between clinical features and early post-RFA recurrence, and the results showed that the model had an AUC of 0.69. However, the predictive performance of the SVM model may decrease when a large number of variables are inputted. Therefore, it is essential to select the variable prior to establishing the SVM model. Conversely, the ANN algorithm cannot be used to select variables, while the key advantage of the ANN model is that it can process data with a large number of variables and samples. In the study of Wu et al. [70], a total of 15 variables were inputted into two ANN models for the prediction of 1-year disease-free survival (DFS) and 2-year DFS, and the performances of these two models were both excellent, with AUCs of 0.964 and 0.974, respectively. Unfortunately, both SVM and ANN models are immature because they lack external validation. The relevant papers are listed in Table 4.

**Table 4:** Characteristics of ML-based predictive model after RFA

Author	Study type	No. of patients	Modality	Model	Outcomes	AUC	Conclusion
Liang [69], 2014	Retrospective Single center	83	US guided	SVM	RR	0.69	The SA + RF SVM method had the best accuracy for predicting high-risk recurrent patients
Wu [70], 2017	Retrospective Single center	431	CT guided	MLP	1,2-year DFS	D: 0.94 (1-year) 0.88 (2-year) V: 0.77 (1-year) 0.72 (2-year)	The MLP-based model with 15 clinical HCC relevant features achieved satisfactory prediction performance for 1-year DFS

*ML* machine learning, *RFA* radiofrequency ablation, *AUC* area under the curve, *US* ultrasound, *SVM* support vector machine, *RR* recurrence rate, *SA* simulated annealing algorithm, *RF* random forest, *CT* computed tomography, *MLP* multilayer perceptron, *DFS* disease free survival, *D* development cohort, *V* validation cohort

### TACE

Most HCC patients are typically diagnosed at intermediate or advanced stages when curative treatments cannot be applied [71, 72]. According to the Barcelona Clinic Liver Cancer (BCLC) staging system [73] and several treatment guidelines [6, 7], TACE is the gold standard for patients with intermediate-stage HCC. Since not all HCC patients can benefit from TACE [74, 75], a predictive model providing therapeutic outcome estimation prior to the procedure is urgently needed for clinical decision making.

Previous studies have constructed a variety of ML models with clinical and radiological variables for predicting the therapeutic outcome of HCC patients after TACE [25, 76–79]. Mähringer-Kunz et al. [76] used traditional imaging features, such as tumor size and tumor number, and other clinical variables to create an ANN model for predicting 1-year survival after TACE. Further, the results demonstrated that the predictive performance of this model was 0.77 in the training cohort and 0.83 in the validation cohort. However, imaging features are not always visible to the naked eye, and some tiny imaging features may be overlooked. Radiomics is an emerging discipline that can extract invisible imaging features such as statistic, shape and texture features from medical images. Abajian et al. [77] established an RF model and used semiautomatic 3D tumor segmenting software to extract several statistic and shape features from MR imaging. The results of their study demonstrated that the most valuable predictor of treatment response following TACE was relative tumor signal intensity on pre-TACE MR images, and the highest predictive accuracy of the RF model achieved 78%. However, the quantitative imaging features in Abajian’s study are too simple and cannot provide adequate information to predict the therapeutic outcome. Liu et al. [78] developed an SVM model with complex radiomic features. These complex radiomic features were first extracted by manual segmentation from static B-mode images, which included 181 statistic features, 13 tumor shape features, and 740 texture features. After extraction, the meaningful radiomic features were selected by the gradient boosted regression trees (GBRT) algorithm [78], and finally, the SVM model was established with an AUC of 0.81 in the internal validation cohort. Indeed, radiomic features can not only be extracted by manual or semiautomatic segmentation tools but can also be extracted automatically by CNN algorithms [25, 78, 79]. Morshid et al. [79] used a CNN-based segmentation protocol to extract a large number of shape and texture features from portal venous phase CT images. Based on these imaging features, an RF model was established, and the results showed that the RF model could accurately distinguish TACE-refractory patients with an AUC of 0.7331 [79]. In addition to extracting imaging features, the CNN algorithm can also be used to establish the predictive model [25, 78]. Peng et al. [25] used the CNN algorithm to automatically extract the imaging features of HCC from CT images and established a predictive model of tumor response after TACE. Their study showed that the CNN models could predict the complete response (CR), partial response (PR), stable disease (SD) and progressive disease (PD) of HCC lesions with AUCs of 0.97, 0.96, 0.95, and 0.96, respectively. Similarly, Liu et al. [78] developed a CNN model to extract imaging features from dynamic contrast-enhanced ultrasound (CEUS) images and predict the objective response of 130 HCC patients after TACE with an AUC of 0.93. The relevant papers are listed in Table 5.

**Table 5:** Characteristics of ML-based predictive model of HCC patients after TACE

Author	Study type	No. of patients	Model	Outcomes	AUC	Conclusion
Abajian [77], 2018	Retrospective Single center	36	RF	Responders or non-responders	–	RF model combined with MRI parameters may be predicted tumor response of post-TACE HCC
Morshid [79], 2019	Retrospective Single center	105	RF	TACE-susceptible or TACE-refractory	0.733	The accuracy of RF model using a combination of clinical parameters plus quantitative image features was higher than the RF model based on the clinical parameters alone, in the study of predicting HCC response to TACE
Mähringer-Kunz [76], 2020	Retrospective Single center	282	ANN	1-year survival	V: 0.77 ± 0.13 D: 0.83 ± 0.06	The ANN model had a promising performance at predicting HCC patient survival after TACE and outperformed the traditional scoring systems
Peng [25], 2020	Retrospective Multicenter	798	CNN	CR, PR, SD, PD	D: 0.97 (CR) 0.96 (PR) 0.95 (SD) 0.96 (PD) V: 0.98 (CR) 0.96 (PR) 0.95 (SD) 0.94 (PD)	The CNN model presented a good performance for predicting the outcome of TACE
Liu [78], 2020	Retrospective Single center	138	CNN SVM1* SVM2#	ORR	D: 0.98 (CNN) 0.84 (SVM1) 0.82 (SVM2) V: 0.93 (CNN) 0.80 (SVM1) 0.81 (SVM2)	CNN is better in predicting treatment response over SVM in HCC patients treated with TACE

*SVM1: radiomics-based time-intensity curve of CEUS model using SVM; ^#^SVM2: radiomics-based B-Mode images model using SVM

*ML* machine learning, *HCC* hepatocellular carcinoma, *TACE* transarterial chemoembolization, *AUC* area under the curve, *RF* random forest, *MRI* magnetic resonance imaging, *ANN* artificial neural network, *D* development cohort, *V* validation cohort, *CNN* convolutional neural network, *CR* complete response, *PR* partial response, *SD* stable disease, *PD* progressive disease, *SVM* support vector machine, *ORR* objective response rate, *CEUS* contrast-enhanced ultrasound

### Sorafenib

Sorafenib is the standard treatment for advanced-stage HCC. The median OS of sorafenib-treated HCC was 10.7 months and 6.5 months in two previous representative randomized controlled trials [80, 81]. Because of the high cost and modest efficacy, a reliable predictive tool is necessary to assist clinicians in adjusting the daily management of sorafenib for such patients.

ML methods are not routinely used for predicting therapeutic outcomes in the treatment of sorafenib for HCC. Choi et al. [22] collected clinical and radiological data from 480 sorafenib-treated patients, and the important variable scores were used to select final parameters based on the RF algorithm. They found that the established model had a better predictive performance in time to progression (TTP) and overall survival (OS) than those of the Child–Pugh and Model for End-Stage Liver Disease (MELD) scores (0.746 vs 0.686 and 0.545 for TTP, 0.875 vs 0.777 and 0.682 for OS). However, this study lacks independent external validation. The relevant papers are listed in Table 6.

**Table 6:** Characteristics of ML-based predictive model of HCC patients after treatment of sorafenib

Author	Study type	No. of patients	Model	Outcomes	C-index	Conclusion
Choi [22], 2017	Retrospective Single center	480	LR	TTP and OS	D: 0.669 (TTP) 0.809 (OS) V: 0.746 (TTP) 0.875 (OS)	The prognostic factors selected by RF algorithm were used to develop an excellent predictive model by LR approach for the prediction of therapeutic outcome after sorafenib

*ML* machine learning, *HCC* hepatocellular carcinoma, *LR* logistic regression, *TTP* time to progression, *OS* overall survival, *D* development cohort, *V* validation cohort, *RF* random forest

### Future perspectives in ML for the prognostic study of HCC

Currently, the ML model for predicting the therapeutic outcome of HCC is usually based on multivariate predictors, such as demographic, clinical, radiological, pathologic and genetic parameters. Selecting the final predictors is a considerable challenge in traditional statistical models because traditional statistical methods may lose some important information. The ML model can include more variables, and it may become a promising protocol over the traditional statistical model. In addition, the ML algorithm can extract and select radiomic features that are invisible to the naked eye, and those novel variables may provide promising predictive value compared with simple radiological parameters (tumor size and tumor number, etc.).

The most important challenge in the ML approach is the accurate selection of algorithms to create the predictive model with external validation for the model. On the one hand, certain types of ML models are favored for specific types of data, such as CNNs for imaging data and SVMs for small sample size data. The ML model should be selected by a thoughtful study design. On the other hand, as there is a need for clinical reality in the future, appropriate external validation should be used to confirm the generalization ability. Due to the lack of a commonly accepted design of ML predictive models for the prognostic study of HCC, it may be possible that the current ML model is not the best one available.

## Conclusion

ML algorithms can automatically extract imaging features and identify optimal subsets of features from large data sets, particularly when combined with radiomics analysis. Relative to traditional statistical models, ML models demonstrate improved predictive performance in the prognostic study of HCC. Regrettably, most existing ML predictive models lack external validation, which is an obstacle to serving HCC patients as personalized predictive tools. Although most current ML algorithms are preliminary, this promising method will be widely accepted in clinical practice in the future.

## Data Availability

Not applicable.

## Abbreviations

AUC: Area under curve
ANN: Artificial neural network
BCLC: Barcelona Clinic Liver Cancer
BN: Bayesian network
CR: Complete response
CPT: Conditional probability table
CEUS: Contrast-enhanced ultrasound
CNN: Convolutional neural network
DT: Decision tree
DNN: Deep neural network
DFS: Disease-free survival
GBRT: Gradient boosted regression trees
HCC: Hepatocellular carcinoma
IHC: Immunohistochemistry
LT: Liver transplantation
LR: Logistic regression
ML: Machine learning
MWA: Microwave ablation
MELD: Model for End-Stage Liver Disease
MLP: Multilayer perceptron
OS: Overall survival
PR: Partial response
PD: Progressive disease
RFA: Radiofrequency ablation
RF: Random forest
RSF: Random survival forests
RNN: Recurrent neural network
SD: Stable disease
SVM: Support vector machine
TTP: Time to progression
TACE: Transarterial chemoembolization
WSI: Whole slide image
